# Z-Plasty technique in congenital midline cervical cleft; a rare case report & literature review

**DOI:** 10.3389/fsurg.2025.1660354

**Published:** 2025-10-06

**Authors:** Ghaith Adi, Felicitas Eckoldt, Ilmi Alhussami

**Affiliations:** 1 College of Medicine, Alfaisal University, Riyadh, Saudi Arabia; 2 Department of Pediatric Surgery, Universitätsklinikum Jena, Jena, Germany

**Keywords:** congenital midline cervical cleft, Z-plasty reconstruction, neonatal neck anomalies, pediatric plastic surgery, branchial arch malformations, genetic associations in CMCC

## Abstract

Congenital midline cervical cleft (CMCC) is a rare developmental anomaly of the anterior neck, often misdiagnosed due to its similarity to other cervical malformations. It results from impaired midline fusion of the branchial arches, leading to a linear skin defect with a fibrotic cord and, in some cases, a sinus tract. Left untreated, CMCC can cause progressive contracture, restricted neck mobility, and aesthetic deformities. This review examines the embryological basis, clinical presentation, histopathological characteristics, differential diagnosis, and surgical management of CMCC, with a focus on Z-plasty as the preferred reconstructive technique. Z-plasty effectively lengthens the scar, prevents recurrent contracture, and restores normal neck contour. In addition, we present a case of a 3-day-old female neonate with CMCC, successfully treated with Z-plasty reconstruction, reinforcing the importance of early intervention. Emerging genetic research suggests a potential hereditary component in CMCC, warranting further investigation into its molecular underpinnings. Advances in regenerative medicine and surgical innovation may improve treatment outcomes, offering new possibilities for personalized management of congenital cervical anomalies.

## 1 Introduction

Congenital midline cervical cleft (CMCC) is a rare congenital anomaly of the anterior neck, with sporadic occurrences reported in the literature (1, 2). It is characterized by a thin, slender, atrophic midline fissure in the skin, underlain by a subcutaneous fibrous cord that creates a soft tissue protuberance (1, 3, 4). Clinically, CMCC presents as a vertically oriented erythematous strip with fluid exudation and, in some cases, mucoid discharge from a blind-ended sinus (1, 3, 4). This sinus extends between the mandibular symphysis and the suprasternal notch (1, 3, 4). CMCC may also be associated with additional developmental anomalies, such as bifid mandible and microgenia (1).

The etiology of CMCC is hypothesized to originate from a failure of midline fusion of the branchial arches during embryonic development, disrupting the normal migration of mesodermal cells from the developing tongue to the ventral neck (3, 5). This aberrant migration results in the formation of a complex unique composite structure composed of skin, skeletal muscle, fibrous tissue, and exocrine elements (3, 5). Although CMCC is an uncommon condition, it can significantly impact a patient's quality of life if left untreated (6). Progressive complications may include neck extension impairment, microgenia, exostosis, torticollis, and recurrent infections, underscoring the importance of early diagnosis and surgical intervention (6).

The management of CMCC requires complete excision of anomalous tissue to prevent the formation of cicatricial contractures and associated morbidities over time (2, 6, 7). However, simple linear closure technique should be avoided, due to its high risk of hypertrophic scarring and recurrent neck contracture (2, 6, 7). Instead, Z-plasty closure is the preferred surgical technique, which involves the transposition of two triangular skin flaps to effectively elongate a linear scar contracture, reconstruct the cervicomental crease, and reduce contracture formation (6, 7). In this report, we describe a case of CMCC successfully managed by using Z-plasty closure. Furthermore, we provide a comprehensive review of the literature on CMCC and its surgical management.

## 2 Case presentation

**Figure 1:**
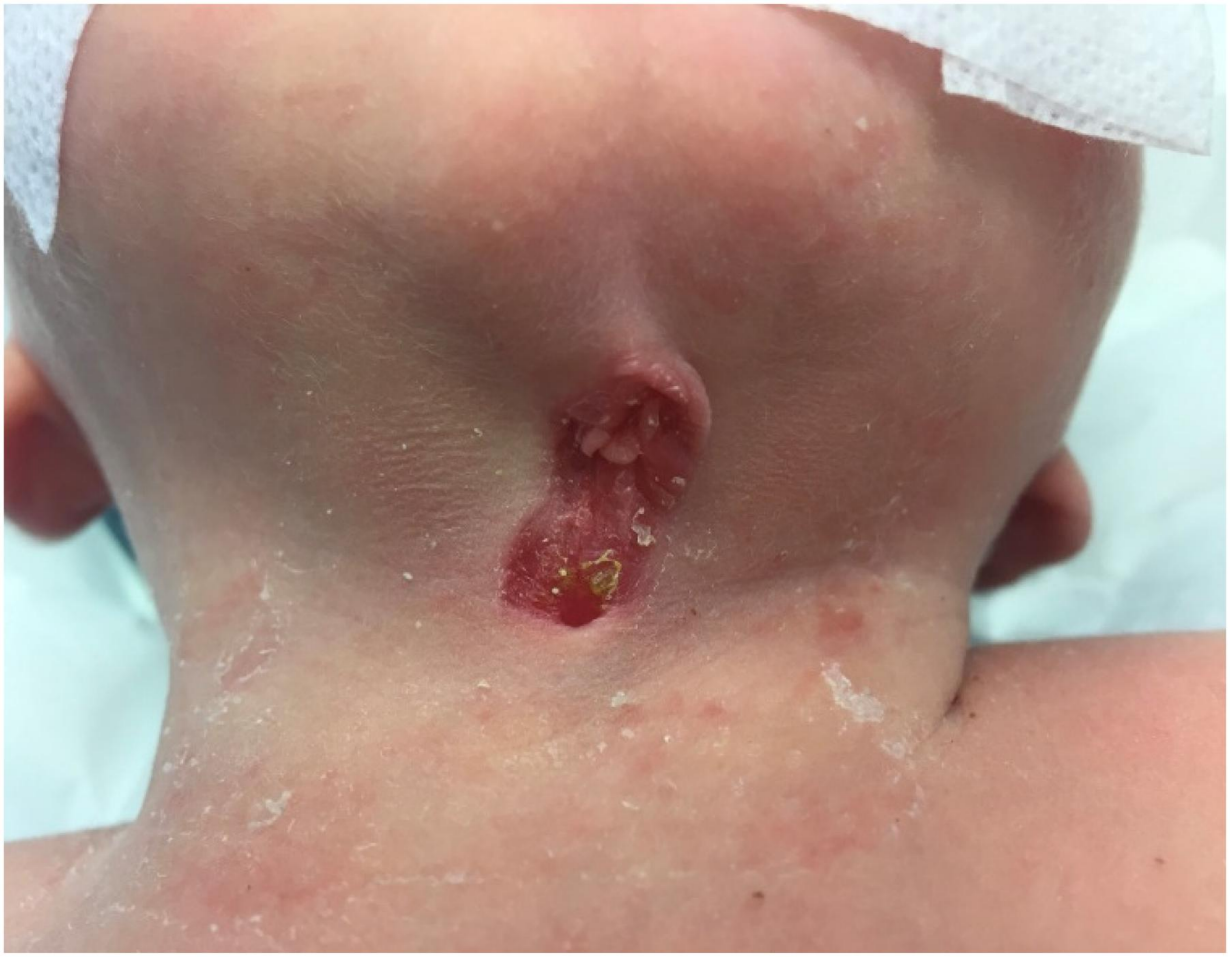
Clinical presentation of the CMCC. The lesion is characterized by erythematous skin, a midline cleft with an upper skin tag, a mucosal sulcus, and a caudal sinus. Neck extension accentuates skin webbing toward the mandible.

We report the case of a full-term, healthy 3-day-old female newborn who was referred to the pediatric surgery clinic due to a congenital anomaly identified at birth. The malformation was localized at the midline of the anterior neck. On examination, a painless lesion measuring approximately 3 cm in length and 1 cm in width was observed. The lesion did not impair breathing or swallowing (Figure 1). It was characterized by an open sinus covered with a mucous membrane, raising suspicion of a potential fistulous tract at its caudal terminus. Notably, the newborn had no other congenital anomalies, and there was no relevant family history. This suggests a non-related genetic predisposition. The lesion remained stationary during tongue protrusion and swallowing, indicating no attachment to the hyoid bone or thyroid gland, both of which were assessed to be structurally normal on physical examination.

**Figure 2:**
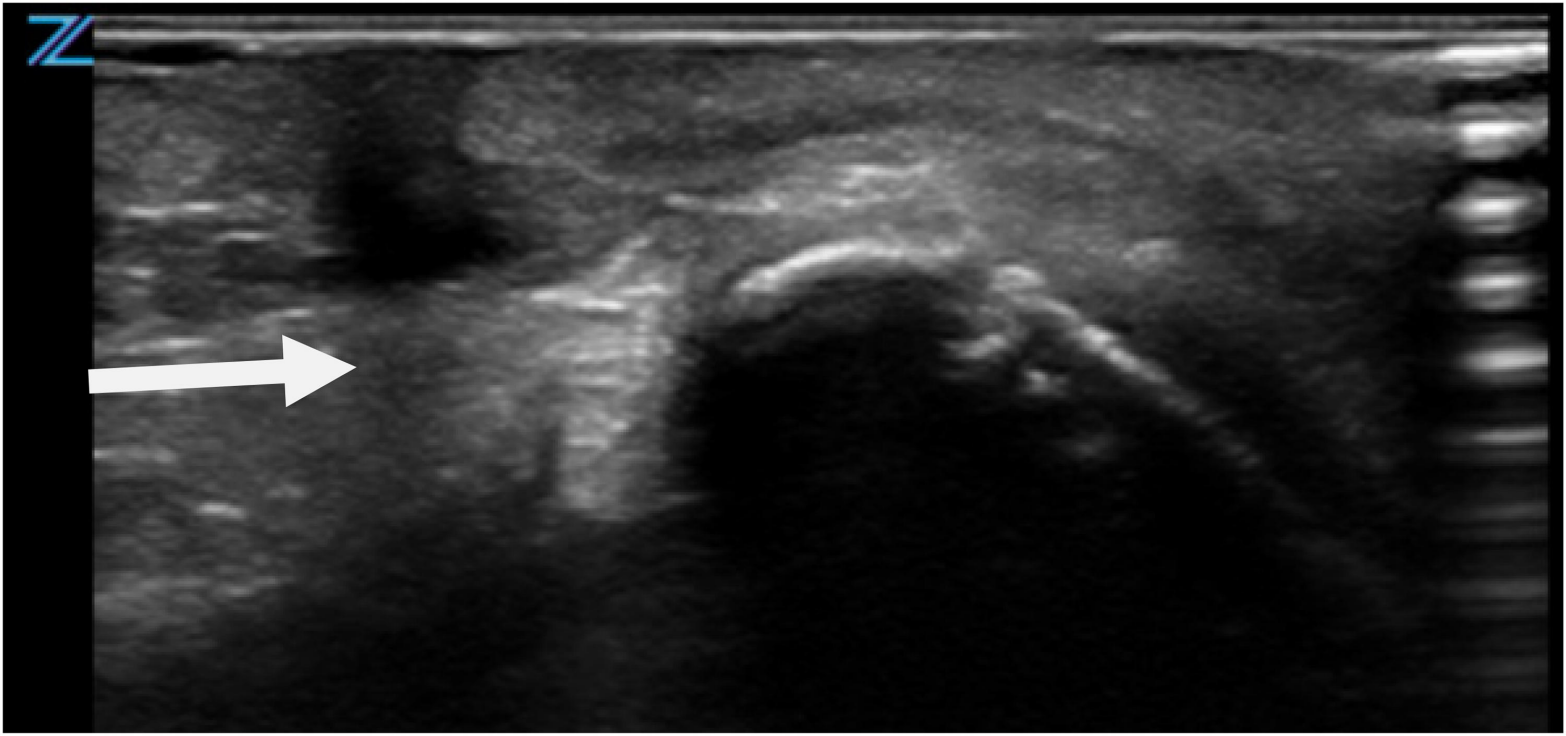
Ultrasonographic evaluation of CMCC. Imaging reveals a blind-ended tract extending caudally, with no evidence of deeper structural involvement.

Given the unusual presentation and potential clinical implications, a comprehensive diagnostic evaluation was initiated. Preoperative clinical assessment revealed normal neck mobility, with no restriction of extension or rotation. Ultrasonography served as the primary imaging modality to explore the lesion's depth and its relationship to surrounding structures (Figure 2). The ultrasound revealed a superficial anomaly with no evidence of deep tissue involvement or communication with adjacent anatomical structures. These findings guided a multidisciplinary team—including specialists from pediatric surgery, neonatology, and pediatrics—to proceed with surgical intervention to fully delineate the extent of the malformation and prevent possible complications.

**Figure 3:**
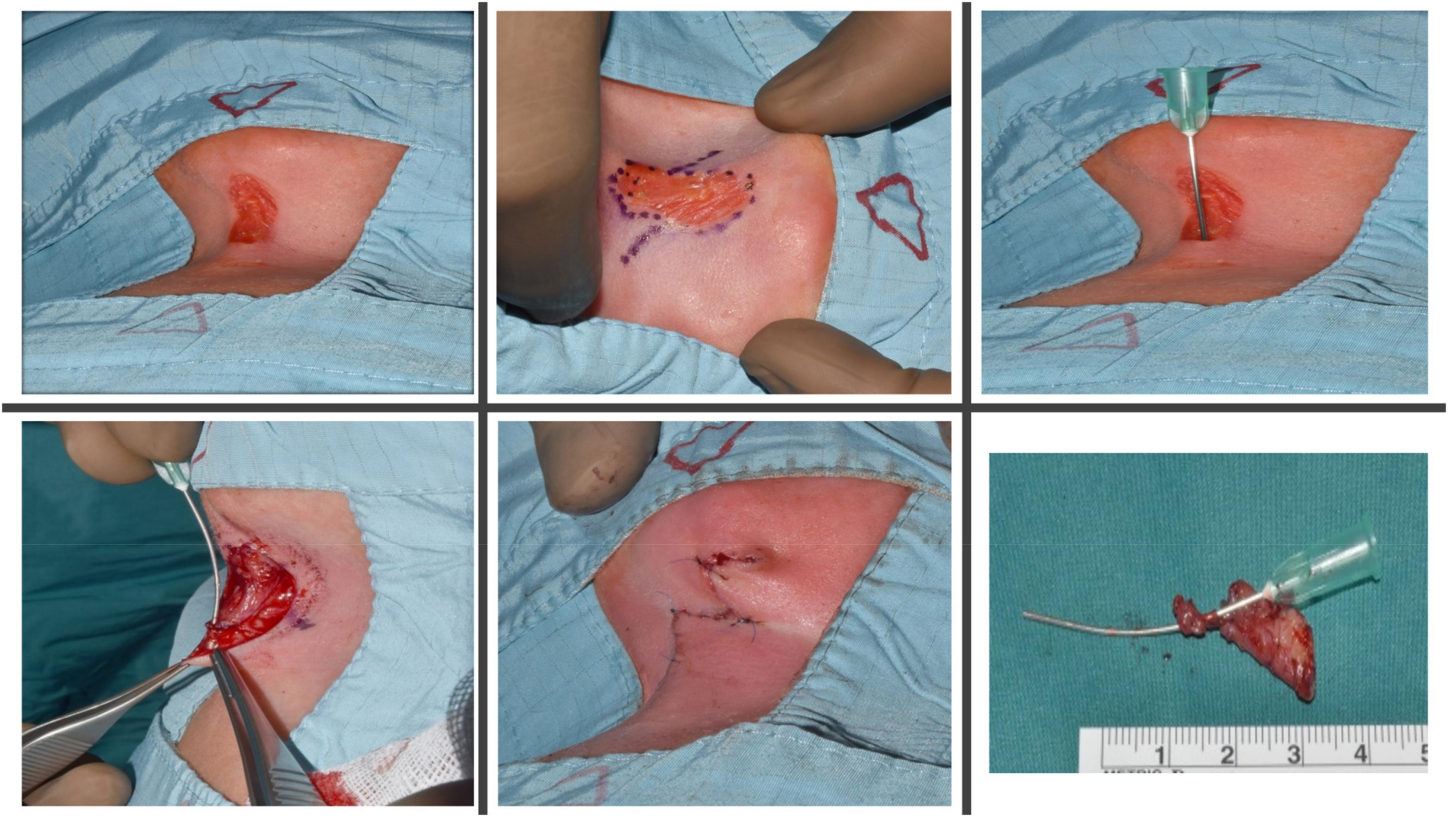
Intraoperative findings and surgical technique. A 60° Z-plasty was performed with an elliptical incision. The upper portion of the lesion was completely excised, while the lower portion, including the fibrotic cord extending to the manubrium, was removed. The resulting Z-plasty flaps were meticulously transposed and sutured in two layers to achieve optimal functional and cosmetic outcomes.

Surgery was performed under general anesthesia with complete excision of the epithelial cleft and underlying fibrous cord. Intraoperative exploration confirmed a CMCC with a caudally positioned duct terminating above the sternum, without extension into deeper structures, thereby excluding the possibility of a complex fistulous tract. A fusiform incision was fashioned, and the specimen was excised *en bloc* to the level of the investing fascia, measuring approximately 3.5–4.0 cm in length. Hemostasis was secured with bipolar cautery. Reconstruction was accomplished using a 60° Z-plasty. The central limb, corresponding to the excised tract, measured 3.5 cm. From each terminus of the central limb, lateral limbs of equal length (3.5 cm) were designed at 60° angles. Triangular flaps were carefully elevated in the subdermal plane, preserving vascularity, and transposed across the axis without undue tension. This maneuver provided effective scar lengthening and reoriented the closure along relaxed skin tension lines. The 60° angle was selected for its reliable balance between flap viability and length gain. Layered closure was performed with 5-0 Vicryl sutures to the dermis and 6-0 Vicryl sutures to the skin. Flaps demonstrated excellent viability with precise inset and alignment. A light sterile dressing was applied. Postoperative recovery was uneventful, with routine wound care and satisfactory healing (Figure 3).

**Figure 4:**
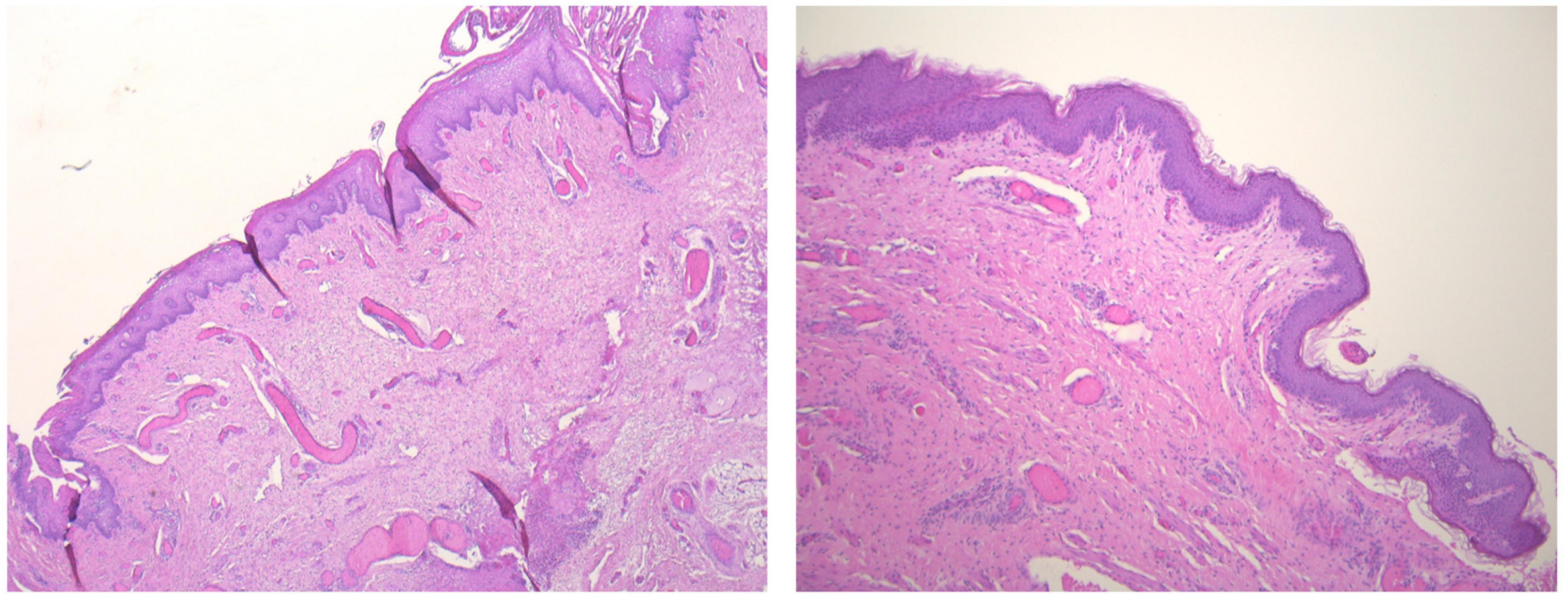
Histopathological features of the CMCC. Hematoxylin and eosin staining (40× magnification) reveals a cleft lined by stratified squamous epithelium with surface parakeratosis. The underlying dermis lacks adnexal structures but contains abundant striated muscle bundles at deeper levels. Similarly, the cephalic papule is composed of a stratified squamous epithelial lining overlying muscle bundles, which progressively transition into normal skin with adnexal structures, highlighting the lesion's interaction with surrounding tissue.

Postoperative histopathological analysis of the excised tissue confirmed the diagnosis of a CMCC, with no evidence of fistulous structures or ectopic salivary gland tissue. These findings were critical in excluding other differential diagnoses and solidifying the classification of the anomaly (Figure 4).

**Figure 5:**
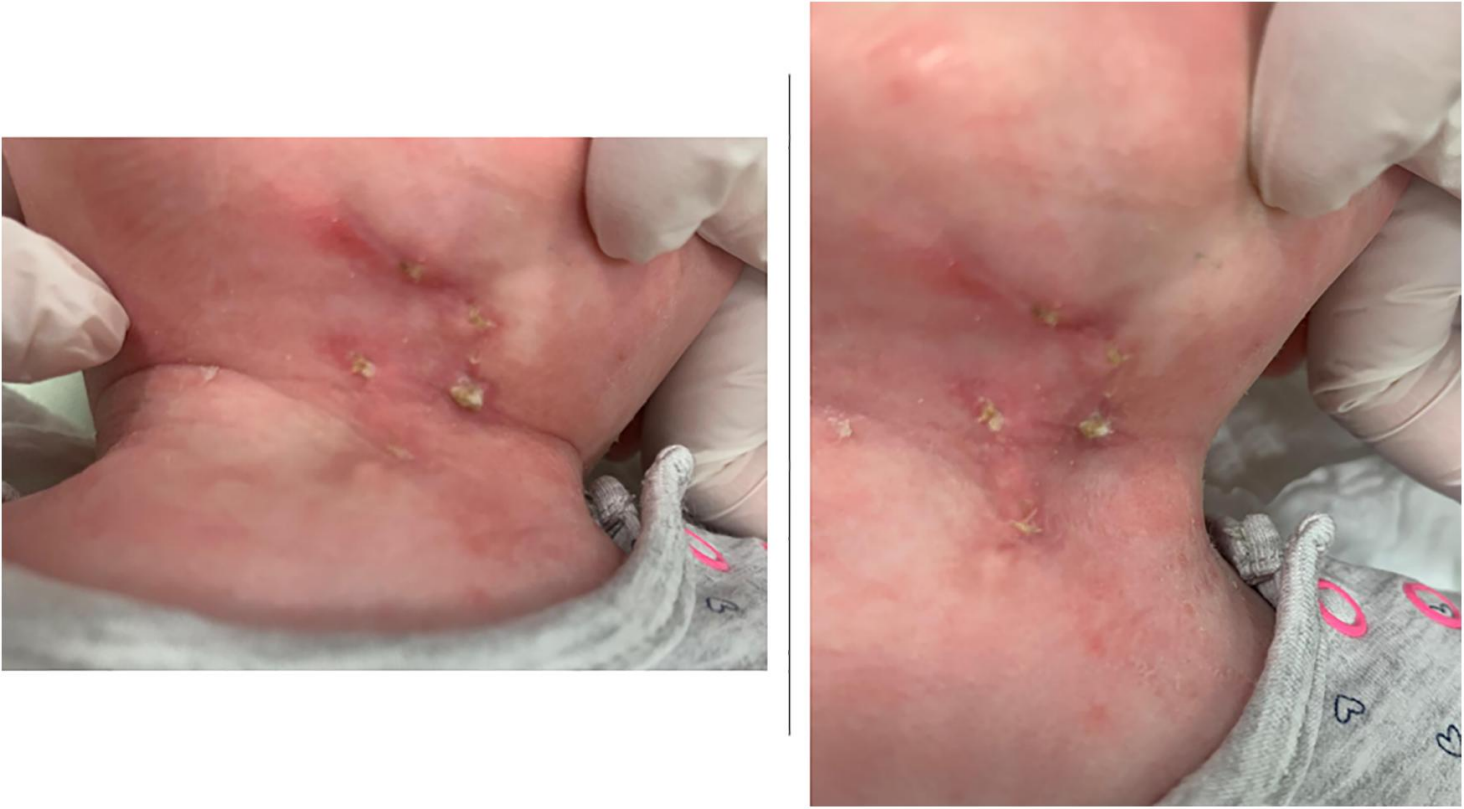
Postoperative wound appearance at 6 weeks follow-up. Clinical photographs of the cervical region showing the surgical site with evidence of partial healing and residual erythema, crusting, and localized inflammatory changes.

**Figure 6:**
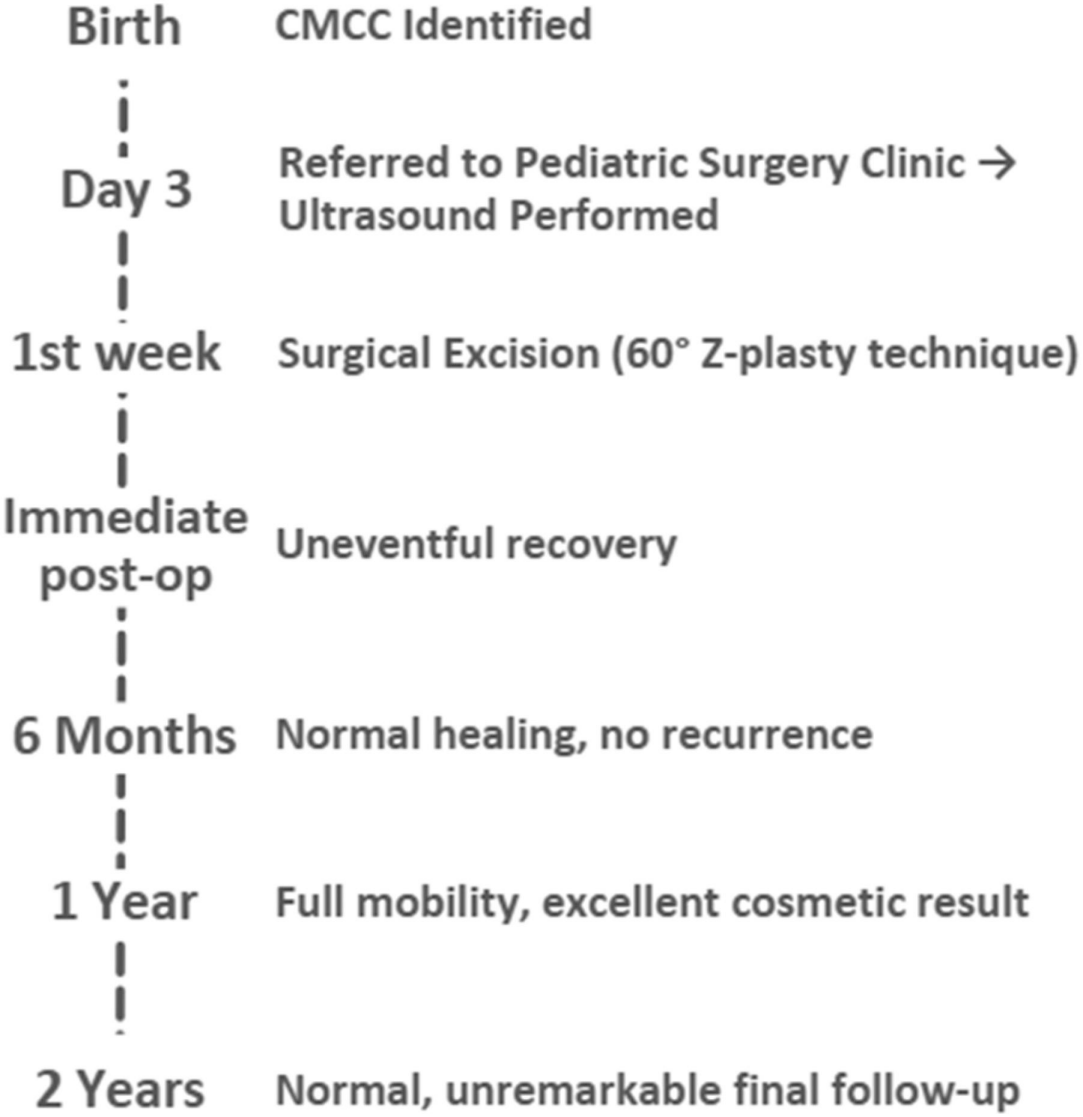
Clinical timeline of a neonatal case of CMCC.

At the 6-week follow-up, clinical examination demonstrated satisfactory wound healing with localized erythema and crusting at the surgical site (Figure 5). The postoperative course was uneventful. Follow-up examinations demonstrated excellent wound healing with no signs of functional impairment or cosmetic disfigurement. The absence of complications or recurrence during the follow-up period underscores the success of the surgical approach and highlights the importance of early intervention in such cases. Chronological timeline of the presented case of CMCC, from birth through 2-year follow-up (Figure 6).

## 3 Discussion

CMCC is a rare congenital anomaly of the anterior neck, first described in the medical literature in the 1940s (2, 3, 6, 8, 9). It has been referred to by various terminologies, including congenital midline cervical cord/cleft, medial cleft, median fissure of the neck, mentosternal dysraphia, and pterygium colli medianum (2, 3, 6, 8, 9). CMCC exhibits a slight female predominance, with an estimated female-to-male incidence ratio of approximately 2:1 (6).

The etiology of CMCC remains poorly understood, necessitating consideration of both genetic and environmental factors (2). Researchers have proposed various hypotheses about their origin. A predominant theory suggests that these clefts represent a spectrum of developmental abnormalities in the branchial arches, originating from a disruption in the fusion between the first and second branchial arches at the midline during embryonic development (2, 6, 9–11). This theory explains the observed variations in the anomaly, which range from a simple cleft-less cord to the complete absence of the thyroid cartilage and hyoid bone (6, 9, 12). The hypothesis suggests that the underlying mechanism for the incomplete branchial fusion is associated with vascular anomalies that leads to ischemia, tissue necrosis, and subsequent scarring (6, 13–17). Other factors that contribute to CMCC development include the persistence of remnants of the thyroglossal duct and sinus cysts, pressure exerted on the cervical area by the pericardial roof, rupture of pathological epithelial adhesions in the first branchial arch between the cardiohepatic fold and the ventral part, and the absence of mesenchymal tissue in the cervical midline (6, 13–17). Additional proposed mechanisms involve aberrant interactions between ectoderm and mesoderm, impaired mesodermal fusion along the distal branchial arches, and defective differentiation of mesenchymal tissue (6, 17, 18). These hypotheses enhance our understanding on the formation of CMCC in the anterior neck.

### 3.1 Genetic contributions to CMCC

Genetic data suggest that CMCC arises from distinct mechanisms in sporadic and familial disease (4, 19). Sporadic cases often harbor heterogeneous variants, such as those in PARD3, MDM4, and EP300, typically inherited from unaffected parents (19). These changes converge on core cellular pathways, supporting a model of polygenic risk with incomplete penetrance rather than a single causal mutation (19). Familial CMCC, in contrast, shows a clearer hereditary signal: truncating variants in TYW1B and SSPO, together with frameshifts in OVGP1, ZAN, and FOLR3, implicate pathways in gamete interaction, embryonic development, and neural organization (4). This divergence underscores the genetic heterogeneity of CMCC, sporadic disease emerging from multifactorial or *de novo* events, and familial disease reflecting pathogenic inheritance (4, 19). Broader sequencing efforts are needed to define whether these represent convergent molecular disruptions or distinct genetic etiologies (4, 19). Table 1 summarizes the current genes and its molecular mechanism in CMCC.

**Table 1:** Reported genes in CMCC, grouped by inheritance pattern with their proposed molecular mechanisms.

Inheritance pattern	Genes	Mechanism	References
Non-related individuals with CMCC	Partitioning Defective 3 (PARD3), Mouse Double Minute 4 (MDM4), Fyn-Related Kinase (FRK), Microcephalin 1 (MCPH1), Fibroblast Growth Factor Receptor 2 (FGFR2), E1A Binding Protein P300 (EP300)	• PARD3 mutation: Disruption of epithelial polarity and neural crest cell migration has been shown to impair palatal midline fusion. • MDM4, FRK, MCPH1, FGFR2, EP300, and PARD3 have been implicated in CMCC, with functions spanning apoptosis inhibition (MDM4), growth suppression (FRK), chromatin remodeling and microcephaly risk (MCPH1), fibroblast growth factor signaling in syndromic craniosynostosis (FGFR2), transcriptional regulation linked to Rubinstein–Taybi syndrome (EP300), and epithelial polarity and cell division (PARD3).	(4, 19, 45–48)
Related individuals with CMCC	Janus Kinase 1 (JAK1), Pregnancy-Associated Plasma Protein A (PAPPA), Zonadhesin (ZAN), tRNA-yW synthesizing protein 1 homolog B (TYW1B), Oviductal glycoprotein (OVGP1), Folate receptor 3 (FOLR3), Subcommissural organ spondin (SSPO)	• AK1, PAPPA, ZAN, TYW1B, OVGP1, FOLR3, and SSPO have also been associated with CMCC, involving interferon signaling (JAK1), proliferative metalloproteinase activity (PAPPA), gamete interaction (ZAN, OVGP1), DNA modification (TYW1B), folate metabolism (FOLR3), and neuronal aggregation in CNS development (SSPO).	(4, 19)

### 3.2 Clinical features and differential diagnosis

CMCC manifests as a variable-length vertical defect, extending from the mandible to the sternum, affecting skin and subcutaneous tissues in the midline of the anterior neck (2, 5, 14, 18, 20). CMCC is histologically characterized by an atrophied epidermal layer with a mucosal surface accompanied by either fibrous connective or glandular tissue (1, 2, 6, 13). Furthermore, CMCC can be associated with the cartilage, skeletal muscle, absence of epithelial adnexa in the dermis, ectopic salivary gland tissue, and pseudostratified ciliated columnar epithelium, particularly in the presence of the sinus tracts (6, 14, 15, 17). At the cephalic end of the anomaly, there is a cleft, nipple-like protrusion, confined to the skin that can extend to the tongue, lip, mandible, and sternum in severe cases (2, 16). The anomaly may also involve a fistula, sinus tract, or duct, which is shallow, blind-ended, and secretes mucous (7, 16, 21). This seromucous secretion is observed to extend towards the manubrium, sternum, or caudally towards the hyoid bone (16, 18, 22). The seromucous discharge tends to resolve spontaneously during infancy (18). A fibrous cord connects the cleft, which appears as a reddish or pinkish linear area with a moist surface and atrophic epidermis, which are the characteristics of CMCC (6, 16).

CMCC diagnosis happens at birth and can be done by physical examination (3, 10). However, correctly diagnosing CMCC can be challenging due to its potential to resemblance to other anomalies since it often appears as a spot-like scar, include branchial cleft anomalies, dermoid cyst, or a thyroglossal duct remnants (Table 2) (2, 3, 6, 17). CMCC may occur as an isolated anomaly or coexist with various conditions, including ectopic bronchogenic cyst, midline hemangiomas, thyroglossal duct cyst, cleft lip, and cleft mandible (3, 6, 17). Additionally, it can be associated with the absence of the hyoid bone or thyroid cartilage, and congenital heart disease (3, 6, 17). Early recognition of these potential concurrent conditions is crucial for accurate diagnosis and effective management.

**Table 2:** Differential diagnosis of CMCC compared with other congenital neck anomalies, highlighting key clinical, imaging, and management features.

Disorder	Location	Sinus/Fistula	Moves with swallowing	Imaging hallmarks	Characteristics	Management	Ref
CMCC	Strict midline anterior neck (chin → suprasternal)	Inferior blind-ending sinus common; mucous discharge possible	No	US/MRI: superficial linear defect + subcutaneous fibrous cord; blind caudal sinus; no relation to hyoid/thyroid	• Visible at birth (Pathognomonic) • Triad: superior nipple-like skin tag, central erythematous atrophic band, inferior sinus • Progressive neck contracture if untreated	• Complete excision with Z-plasty • Early surgery to prevent contracture	(1–4, 49)
Thyroglossal Duct Cyst	Midline ± paramedian near posterior aspect of the hyoid; anywhere along thyroglossal tract	May form after infection or drainage	Yes It elevates on tongue protrusion and with swallowing	US: anechoic/hypoechoic cyst near hyoid; CT/MRI: close relation to hyoid; confirm orthotopic thyroid (claw sign)	• Usually presents in childhood (not a surface cleft) • Recurrent infections • supra- or infrahyoid location	• Sistrunk procedure	(50–54)
Second Branchial Cleft Anomaly	Lateral neck along anterior border of SCM; tract toward tonsillar fossa Usually unilateralAlthough it can be bilateral	Common — sinus (one opening) or fistula (two openings)	No	US/CT/MRI: well-defined lateral cyst; tract following classic course to tonsillar fossa	• Often later (childhood/adolescence) • Recurrent lateral neck infections; external punctum anterior/medial to SCM	• Complete tract excision	(55–59)
Dermoid/Epidermoid Cyst	Midline submental/floor of mouth; above or below mylohyoid	Rare	No	US: often echogenic contents; CT: fat density/calcifications possible, well-circumscribed mass	• Congenital; often noted later • Doughy, slow-growing, non-tender mass; no tongue movement	• Surgical excision	(54, 60–64)
Lymphatic Malformation (Cystic Hygroma)	Posterior triangle or lateral neck; may be multicompartment and extend to mediastinum Often lateral/multiloculated	No true sinus; may transilluminate; can bleed/infect	No	US/CT: multiloculated cystic mass with thin septations	• Commonly present at birth/infancy • Soft, compressible, transilluminant; airway risk in infants; enlarges with infection/hemorrhage	• Sclerotherapy • Complete excision	(65, 66)

### 3.3 Surgical management and prognosis

The prognosis and management of CMCC significantly depend on their size and location (2). Early detection and surgical intervention are critical to prevent complications (2, 6, 17). If not addressed promptly, the cleft can heal into a longitudinal scar with a fibrous band, leading to cicatricial contracture of the neck, restricting neck movement, and potentially causing complications like micrognathia, mandibular exostosis, or hypoplasia (2, 6, 17). Simple excision followed by a simple straight-line closure may lead to scarring and contracture, making Z-plasty the preferred surgical technique for these clefts (2, 7).

Z-plasty is a widely recognized surgical technique in plastic and reconstructive surgery (2). This procedure involves creating a central limb incision with bilateral limb incisions to form two opposing triangular transposition flaps in a “Z” pattern (23, 24). This technique facilitates the release of scar contracture, alters the direction and length of a contracted scar or defect, allows for tissue mobilization and realignment, reduces skin tension, and enhances soft tissue contour, which makes Z-plasty uniquely valuable in the neck by both preventing recurrent contracture and camouflaging scars within natural skin creases (1, 2, 23, 25–29).

The extent of tissue lengthening in Z-plasty is correlates with the angle between the central and bilateral incisions. Table 3 shows the length of scar resulting from different angles between the limbs in a Z-Plasty procedure, highlighting how different geometries influence lengthening and clinical application (1, 2, 23, 28–30). Larger angles yield more lengthening but increase skin tension, possibly causing tissue distortion and dog-ear deformities (2, 23). Narrower angles ease closure but limit lengthening and increase the risk of flap necrosis due to reduced blood flow to the flap tips (2, 23). A 60° angle in Z-plasty is often ideal for maximizing tissue lengthening while ensuring ease of closure, achieving a 90° scar rotation and a 75% increase in length. However, variations in angles and limb lengths are feasible, leading to “skew Z-plasties” (2, 23, 24, 29). These are particularly useful in situations where anatomical constraints hinder the application of symmetric Z-plasties (2, 23, 24, 29). For angles over 60°, the usage of a “compound Z-plasty” is indicated by splitting the angle into smaller equal flaps to reduce skin deformities with an extra scar (29). For longer scars, “serial Z-plasty” distributes tension by adding multiple flaps along the scar, improving flexibility and outcome (29).

**Table 3:** Z-plasty limb angle with corresponding potential scar length gain and clinical selection guidance.

Angle between the central and lateral limbs	Potential increase in length	Clinical selection guidance
30°	∼ 25%	Minimal gain; Narrow flaps, higher necrosis risk
45°	∼ 50%	Moderate gain; Useful with limited laxity.
60°	∼ 75%	Best balance of gain and viability; Preferred in infants’ thin neck skin.
75°	∼ 100%	Large gain; Higher closure tension, needs lax skin
90°	∼ 120%	Maximal gain; High tip tension, Rarely used in pediatric neck.

Compared with alternative scar-revision strategies, Z-plasty confers distinct advantages that are directly relevant to CMCC (31, 32). W-plasty effectively camouflages linear scars by irregularizing their contour along relaxed skin-tension lines, but it provides no true lengthening and is therefore inadequate when contracture release is required (33, 34). Local flap transfers, such as advancement, rotation, or transposition flaps, can supply well-vascularized adjacent tissue and are useful for larger or composite defects, though they necessitate broader dissection and carry risks of donor-site morbidity and contour irregularities (35–37). By contrast, Z-plasty simultaneously lengthens contracted skin, reorients the central limb into favorable vectors, and breaks the scar into a less conspicuous pattern (29, 31). A 60° design reliably achieves ∼75% lengthening with ∼90° scar rotation, and serial or multiple Z-plasties can emulate the irregularizing effect of W-plasty while still ensuring measurable lengthening (29, 31, 38). For CMCC, most reported series favor excision with Z-plasty closure, as it reliably restores neck extension and cervicomental contour while minimizing recurrent tethering and visible scarring (2, 22).

### 3.4 Regenerative adjuncts to Z-plasty

Emerging regenerative strategies, particularly stem cell–assisted flap transplantation, represent a promising frontier for CMCC repair. Preclinical studies demonstrate that mesenchymal stem cells (MSCs) can enhance skin flap survival and neovascularization thereby improving long-term viability (39–42). More recently, a 2023 large-animal model of cervical skin injury showed that MSCs, particularly when combined with platelet-rich plasma, accelerate re-epithelialization, organize collagen fibers, and limit contraction—hallmarks of regenerative rather than reparative healing (40). Meta-analyses further affirm that MSC–scaffold treatments markedly boost wound closure, angiogenesis, collagen deposition, and growth-factor expression in preclinical burn wound models, and exosome-based MSC derivatives show emerging promise for restoring skin structure and function in reconstructive contexts (39, 43, 44). These mechanisms are directly relevant to CMCC, where atrophic epidermis, subdermal fibrosis, and absent adnexal structures predispose to postoperative scarring and contracture despite meticulous Z-plasty. While CMCC-specific trials are lacking, such data support hypothesis-generating studies exploring biologically augmented flap repair as a complement to surgical technique.

## 4 Conclusion & future directions

In conclusion, CMCC is a rare anomaly that demands early surgical intervention to prevent progressive contracture, functional limitation, and cosmetic deformity. Z-plasty remains the gold standard of repair, offering reliable lengthening and favorable outcomes. The present report, however, is inherently limited by its single-case design, which cannot establish the universality of surgical success or permit comparison across different Z-plasty techniques. Longer follow-up in larger cohorts, ideally through multicenter case-control studies, will be necessary to determine the durability of functional and aesthetic results and to clarify the relative advantages of different flap angles.

The absence of genetic testing in this patient represents another important limitation, as it prevents exclusion of a contributory hereditary factor and underscores the need for systematic molecular investigations. Comprehensive genomic profiling could help define the role of genetic predisposition in CMCC, refine risk stratification, and improve counseling for affected families. Future advances in regenerative medicine—particularly stem cell–assisted flap transplantation and other biologically augmented strategies—may complement conventional Z-plasty by enhancing flap survival, reducing contracture recurrence, and improving long-term scar quality. Rigorous, collaborative studies will be essential to translate these approaches into evidence-based, personalized care for patients with CMCC.

## Data Availability

The original contributions presented in the study are included in the article/Supplementary Material, further inquiries can be directed to the corresponding author.
